# Real-world treatment patterns and unmet needs in spinal muscular atrophy: a caregiver-centric survey study from China

**DOI:** 10.1186/s12883-026-04774-z

**Published:** 2026-02-27

**Authors:** Wenxiang Fan, Wan-Er Zheng, Ji Li, Rui Wang, Shaoqing Ni, Pingping Xue, Chi Xu

**Affiliations:** 1 Department of Clinical Trials Institute Management Office, Children’s Hospital, Zhejiang University School Of Medicine, National Clinical Research Center For Children And Adolescents’ Health And Disease, Hangzhou, 310052 China; 2 Department of Neurobiology and Acupuncture Research, Key Laboratory of Acupuncture and Neurology of Zhejiang Province, The Third Affiliated Hospital of Zhejiang Chinese Medical University, Hangzhou, 310053 China; 3 Department of Reproductive Medicine Center, Changzhou Maternal and Child Health Care Hospital, Changzhou Medical Center, Nanjing Medical University, Changzhou, 213000 China; 4 Hangzhou Medical College, Hangzhou, 310053 China

**Keywords:** Spinal muscular atrophy, Disease-modifying therapy, Caregiver burden, Rehabilitation, Genetic screening

## Abstract

**Objective:**

This exploratory survey study aimed to evaluate the epidemiology, treatment patterns (including pharmacotherapy, rehabilitation, and supportive care), economic burden, drug preferences, and unmet needs of Chinese SMA patients.

**Methods:**

We conducted an anonymous online survey using a self-developed questionnaire from March 4 to March 31, 2024. The questionnaire collected comprehensive data on epidemiology, treatment patterns, rehabilitative interventions, nursing management, economic burden, and preferences. Data analysis was performed using descriptive statistics and chi-square tests.

**Results:**

Analysis of 82 valid questionnaires revealed that 91.5% of SMA patients had type I or II disease, with 69.5% diagnosed by genetic testing within 12 months of birth. Awareness of prenatal screening was low. Nusinersen and Risdiplam showed comparable utilization rates and efficacy, though Risdiplam was associated with significantly higher rates of adverse events. Over 75% of caregivers reported financial constraints, with more than 42% expressing long-term affordability concerns. Rehabilitation participation reached 86.6%, predominantly involving motor function training, stretching therapy, and the use of assistive devices. Critical gaps included the finding that 89.0% of caregivers lacked formal training, 40.8% of patients lacking respiratory interventions, and a high demand for emergency skills training (61.0%). Treatment preferences prioritized oral administration, liquid formulations, and fruit flavors, with efficacy, affordability, and ease of use (80.5%) being the primary decision-driving factors.

**Conclusion:**

The management of SMA in China faces three primary challenges: inadequate prevention awareness, critical caregiving deficiencies, and prohibitively high treatment costs. Urgent actions include scaling up prenatal/newborn screening, establishing multidisciplinary care models to address respiratory/nutritional gaps, and implementing policy reforms to improve affordable drug access.

**Supplementary Information:**

The online version contains supplementary material available at 10.1186/s12883-026-04774-z.

## Introduction

Spinal muscular atrophy (SMA) is an autosomal recessive disease caused by mutations in the SMN1 gene on chromosome 5, leading to reduced expression of the survival motor neuron protein (SMN). This causes dysfunction and degeneration of α-motor neurons in the spinal cord and brainstem, resulting in progressive muscle atrophy and weakness of the limb, trunk, bulbar (which controls swallowing) and respiratory muscles [1]. The global carrier frequency of single-allele SMN1 deletions and pathogenic variants is approximately 1 in 52. The disease incidence is approximately 1 in 12,000 [2], with estimated rates ranging from 1 in 3,900 to 1 in 16,000 across 18 European countries [3] and 1 in 11,000 in the United States [4]. In China, the incidence rate among newborns is approximately 1/9788 [5], and the population carrier frequency is approximately 2% [6]. Historically, SMA has been subdivided into three main pediatric-onset types (Type I to III) and two less common types (Type 0 and Type IV): Type 0 has an onset before birth with respiratory failure at birth and a survival period of less than 6 months; Type I (50–60% of cases), the most severe form, leads to respiratory failure before age two; Type II presents before 18 months, allowing sitting but not independent walking; Type III manifests after 18 months with independent walking ability; and Type IV, the adult-onset form after 18 years, follows a mild course without loss of ambulation or major complications [1, 7]. Notably, a confirmed diagnosis of SMA necessitates lifelong treatment, regardless of type.

The advent of disease-modifying therapies (DMTs), including Nusinersen (an intrathecal antisense oligonucleotide that modifies SMN2 splicing) [8], Risdiplam (an oral SMN2 splicing modifier), and onasemnogene abeparvovec [9] (an intravenous gene therapy that delivers a functional copy of the SMN1 gene [10]), has transformed SMA from a relentlessly progressive condition to a treatable disorder [11]. The inclusion of Nusinersen (2022) and Risdiplam (2023) in China’s National Reimbursement Drug List (NRDL) led to a marked improvement in treatment accessibility, as their prices decreased substantially from RMB 700,000 to RMB 33,000 per dose and from RMB 63,800 to RMB 3,780 per bottle, respectively. Furthermore, in several of China’s 34 provincial-level administrative divisions, secondary reimbursement mechanisms, such as critical illness insurance, are available to further lower patients’ out-of-pocket expenditures. Meanwhile, onasemnogene abeparvovec remains in clinical trials and is not yet marketed in China. A recent study indicates that in 2023, basic medical insurance covered 49% of direct medical costs (32% of total costs), yet patients still paid 25.73% of the total cost out-of-pocket for these direct expenses [12]. Clinical trials such as ENDEAR (Nusinersen) and FIREFISH (Risdiplam) demonstrated unprecedented efficacy in halting functional decline and achieving milestone attainment [8, 13]. However, pivotal trials primarily enrolled genetically homogeneous cohorts under stringent protocols, limiting the generalizability of their findings to real-world populations with diverse socioeconomic backgrounds, comorbidities, and treatment access barriers.

In China, access to DMTs for patients with (SMA is primarily achieved through three channels: hospitals, pharmacies, and clinical trials. Hospitals serve as the primary and most regulated avenue for DMTs listed in (NRDL. For agents not stocked in hospital formularies, such as Risdiplam, an oral medication, patients can obtain subsequent supplies from designated pharmacies with a valid prescription after the initial hospital visit or administration. Furthermore, investigational or post-marketing (Phase IV) drugs are accessible free of charge to eligible participants through clinical trial programs. While the efficacy of DMTs is well-established, patient-centered treatment experiences, particularly caregiver-reported outcomes on treatment burden (e.g., burden of intrathecal injections), safety, tolerability, and quality-of-life impacts, remain inadequately characterized. Moreover, although real-world evidence on SMA is accumulating globally [14], comprehensive studies within the specific context of China’s healthcare system, reimbursement environment, and care models remain scarce. This study aims to address this gap by examining DMT utilization patterns and caregiver-reported experiences in a resource-limited setting. Beyond pharmacotherapy, comprehensive SMA management necessitates integrated multidisciplinary support encompassing rehabilitative, nutritional, and respiratory interventions [15, 16]. However, systematic quantification of these unmet needs is still lacking.

This exploratory study, drawing on insights from 82 caregivers, provides an initial, multi-dimensional profile of real-world treatment patterns among Chinese SMA patients. While prior research, such as systematic reviews on DMT efficacy and safety in Asian populations [17], has addressed specific therapeutic aspects, this work extends the focus beyond pharmacotherapy to encompass rehabilitation practices and nursing care. It identifies key unmet clinical needs from the patient and caregiver perspective and offers preliminary evidence to inform the development of patient-centered care models, supportive health policies, and tailored clinical research frameworks.

## Methods

### Study design

This study specifically targeted caregivers of individuals clinically diagnosed with SMA. Eligible participants were required to be the primary caregiver of an individual with a confirmed SMA diagnosis, to voluntarily consent to participate, and to possess the ability to complete the web-based survey.

### Questionnaire development and validation

The study utilized a structured, self-administered questionnaire developed specifically for this study. The design process followed an iterative, multi-step approach to ensure content relevance, clarity, and feasibility for the target population.

#### Item generation and initial development

The initial item pool was developed based on published literature or established consensus, spanning the multidisciplinary management of SMA, rehabilitation practices, current therapeutic research, patient-reported outcomes, and areas of unmet needs [11, 15, 18–21]. Specifically, the survey assessed demographic characteristics (including those of the patients and caregivers), current medical treatments, rehabilitative intervention utilization, nursing management, clinical trial engagement perceptions, and medication needs and preferences.

#### Content validity and expert review

To ensure the questionnaire’s relevance and appropriateness for the Chinese SMA caregiver context, the initial draft underwent sequential rounds of qualitative review by key stakeholders. First, the draft was reviewed by a small group of SMA patient caregivers (*n* = 2) and the corresponding author (Wenxiang Fan)’s field experience in SMA clinical studies. Feedback focused on the comprehensibility of items, the appropriateness of response options, and the identification of any missing key issues from the caregiver perspective. Second, the revised questionnaire was then submitted for expert review. It was evaluated by SMA clinical neurologist, and by specialists from the Meier Advocacy and Support Center (MASC), China’s foremost SMA patient advocacy organization. Their input ensured clinical accuracy, cultural relevance, and that the domains covered the full spectrum of real-world management challenges.

#### Pilot testing and final refinement

A pilot test was conducted to assess feasibility and validity. The final online version was professionally programmed, tested, and formally deployed online. To avoid repeated submissions, designated personnel conducted data verification, and each account was limited to a single response. The full version of the questionnaire can be found in the supplementary document.

### Questionnaire design

#### Demographic characteristics

Patient demographic characteristics included gender, genetic testing, age of diagnosis, SMA type, symptoms before diagnosis, current motor function, comorbidities, medical treatments, and rehabilitation treatments. The demographic characteristics for the caregivers included geographic regions, relationship to the child, awareness of genetic testing, and genetic testing during pregnancy.

#### Current medical treatments

Caregivers were systematically queried regarding patients’ current therapeutic regimens, with focused investigation on the utilization patterns of two approved DMTs: Nusinersen and Risdiplam. The structured assessment encompassed hospital availability of medications, medication access channels, dosage, therapeutic response metrics, adverse reactions, perceived medication efficacy, medication price, financial burden analysis, and implementation challenges.

#### Rehabilitative intervention utilization

Caregivers were asked about rehabilitative intervention utilization, including locations, exercise training, stretching training (including the use of assistive devices), respiratory and sputum management, and swallowing and nutrition management.

#### Nursing management

Caregivers were asked about nursing management provided to the patients. This section assessed the Perceived Importance of Nursing, the receipt of nursing training, the content of such training, the channels through which nursing knowledge was acquired, and caregivers’ specific needs in the field of nursing.

#### Clinical trial engagement perceptions

We investigated caregivers’ perspectives on clinical trials, including awareness of ongoing SMA drug trials, willingness to enroll their child, and perceived role of clinical trials in SMA treatment.

#### Medication preferences and economic considerations

This section assessed preferred administration routes, optimal dosage forms, palatability requirements, and affordable pricing. Additionally, caregivers were asked about the ideal drug characteristics.

### Recruitment and data collection

Participants were invited to complete an online questionnaire. Recruitment followed a non-probability, convenience sampling approach. The survey link was disseminated through social platforms (public platforms and WeChat groups composed with targeted patient by using the “WeChat” applicaion). The survey was administered anonymously to encourage candid responses. To prevent duplicate entries, the survey platform was configured to allow only one response per unique account (tied to a mobile phone number for verification). These “WeChat” groups are maintained by patient advocacy organizations, with an estimated total membership of approximately 1,000 individuals. No paid advertising was used. Due to the closed nature of these groups, traditional metrics such as advertisement “impression” counts are not applicable. The 73-item instrument incorporated adaptive skip-logic patterns that tailored question sequencing to caregivers’ specific circumstances, resulting in variable completion paths, meaning not all items were answered by every participant (supplementary document). No time constraints were imposed during questionnaire completion. The survey spanned a 28-day period from March 4 to March 31, 2024, ensuring temporal consistency in data acquisition while accommodating participants’ scheduling constraints.

### Ethic approval and informed consent

As described in the “Ethics declarations” section, the requirement for physical signatures was waived. Instead, a digital consent mechanism was implemented. Participants first reviewed a written information statement explaining the study’s purpose and procedures. To proceed, they were required to actively select “Agree” to enter the questionnaire; otherwise, access was denied. This procedure ensured that explicit digital consent was obtained from every participant strictly prior to data collection.

### Data analysis

Data were extracted from the survey platform (“Wenjuanxing”) to Excel and checked by professional. Data quality was assessed prior to analysis. Questionnaires were considered incomplete and excluded if: (a) implied consent was not provided, or (b) over 50% of the core demographic and clinical variables (e.g., SMA type, age at diagnosis, treatment status) were missing. Categorical data are presented as n (%) with open-ended responses thematically summarized. Statistical analyses were performed using SPSS 20.0. The primary statistical comparison was between the two main disease-modifying therapy groups. Categorical variables for users of Nusinersen versus users of Risdiplam were compared using Pearson’s chi-square test. For cases where the assumptions of the chi-square test were not met (specifically, when more than 20% of cells had an expected count of less than 5, or for 2 × 2 tables with any expected count < 5), Fisher’s exact test was employed. A two-tailed *p*-value < 0.05 was considered statistically significant.

## Results

### Characteristics of patients and their caregivers

#### Characteristics of patient caregivers

The final study cohort comprised 82 caregivers, representing 23 provincial regions of China (Supplementary Fig. S1). Caregivers were predominantly mothers (84.1%, 69/82). Rehabilitation challenges were the most frequently reported treatment barrier (91.5%, 75/82), followed by high medical costs (82.9%, 68/82) and limited medication accessibility (62.3%, 51/82). In contrast, awareness of genetic testing was relatively low (15.9%, 13/82) (Table 1).

**Table 1:** Characteristics of caregivers (*n* = 82)

Characteristics	Categories	Records [*n* (%)]
Relationship	Father	8 (9.8)
	Mother	69 (84.1)
	Others	5 (6.1)
Challenges in the treatment of SMA	difficulty in diagnosis	41 (50.0)
	limited availability of medications	51 (62.3)
	high medical costs	68 (82.9)
	challenges in rehabilitation therapy	75 (91.5)
	Others	4 (4.9)
Awareness of genetic testing	Yes	13 (15.9)
	No	69 (84.1)
Prenatal genetic screening	Yes	2 (15.4)
	No	11 (84.6)

#### Characteristics of patients

**Fig. 1:**
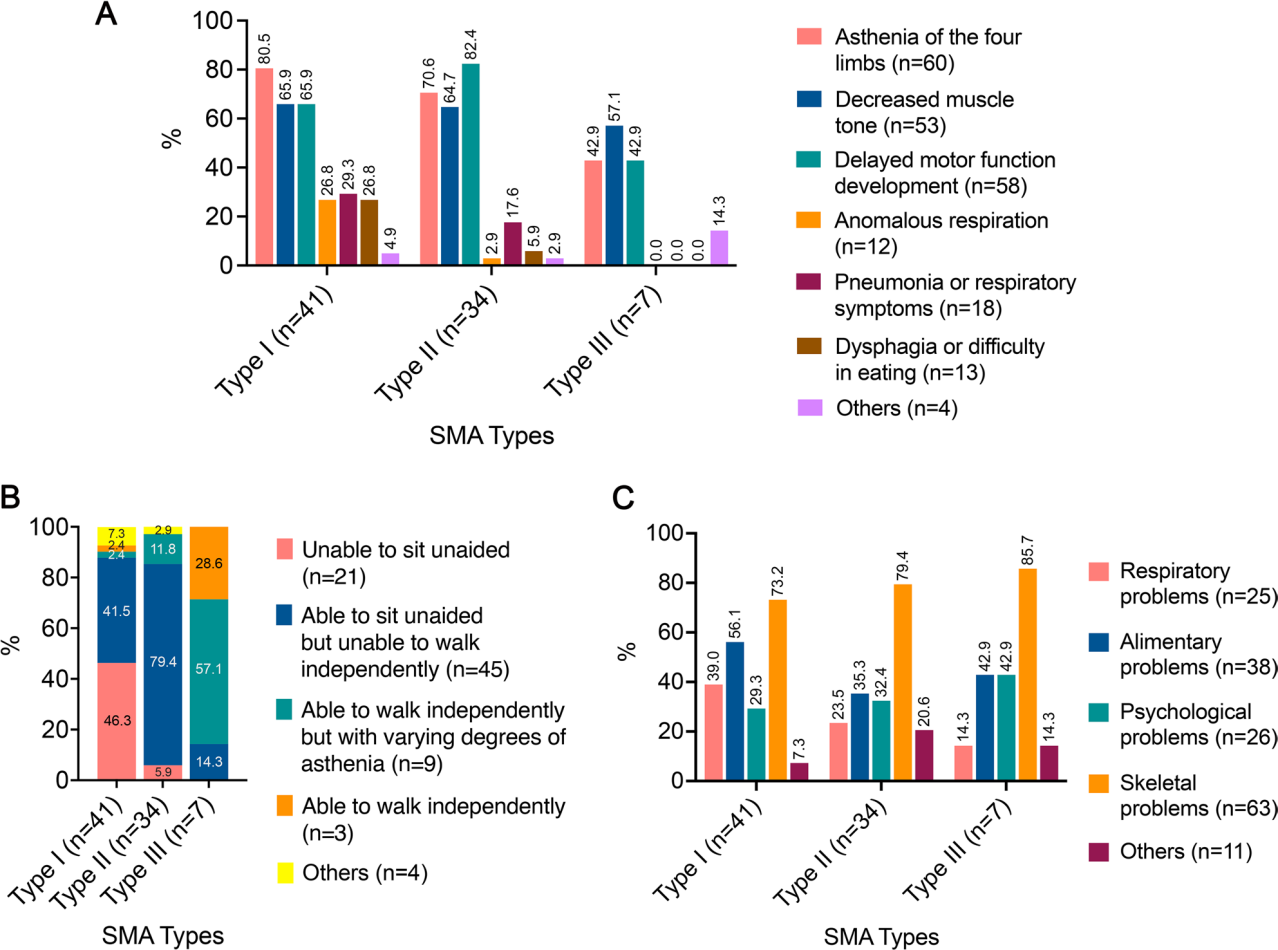
Distribution of participants symptoms before diagnosis, current motor function and other problems by SMA type. **A** Percentage of responses stratified by patient symptoms before diagnosis; **B** Percentage of responses stratified by patient current motor function; **C** Percentage of responses stratified by patient other problems and SMA type. Histograms represent the percentage of participants in each symptom before diagnosis, current motor function and other problems

The demographic and clinical characteristics of the 82 children with SMA are summarized in Table 2. The cohort had a slight female predominance (53.7%, 44/82) and consisted mainly of patients diagnosed before 12 months of age (69.5%, 57/82), with type I SMA being the most common (50.0%, 41/82), followed by type II (41.5%, 34/82) and type III (8.5%, 7/82). The predominant pre-diagnosis manifestations included asthenia of the four limbs (73.2%, 60/82), delayed motor development (70.7%, 58/82), and decreased muscle tone (64.6%, 53/82), which presented distinct patterns across different subtypes of SMA (Table 2; Fig. 1A). Current motor function assessments showed that 25.6% (21/82) of patients never achieved sitting, 54.9% (45/82) could sit but not walk, and 3.6% (3/82) retained walking ability. The motor function profile of each subtype was as follows: Type I was characterized by the failure to achieve sitting (46.3%, 19/41), Type II by the ability to sit but not walk (79.4%, 27/34), and Type III by independent walking with varying degrees of weakness (57.1%, 4/7) (Table 2; Fig. 1B). Pharmacotherapy was received by 96.3% (79/82) of patients and rehabilitation programs were received by 86.6% (71/82). Among the treated patients, skeletal abnormalities were the most prevalent comorbidities (79.7%, 63/79), followed by gastrointestinal disorders (48.1%, 38/79), psychological symptoms (32.9%, 26/79), and respiratory conditions (31.6%, 25/79). The distribution of these comorbidities varied across SMA types (Table 2; Fig. 1C).

**Table 2:** Characteristics of patients (*n* = 82)

Characteristics	Categories	Records [*n* (%)]
Gender	Male	38 (46.3)
	Female	44 (53.7)
Genetic testing	Yes	82 (100.0)
	No	0 (0.0)
Age of diagnosis	<6 Months	30 (36.6)
	6–12 Months	21 (25.6)
	12–18 Months	14 (17.1)
	≥ 18 Months	17 (20.7)
SMA type	Type I	41 (50.0)
	Type II	34 (41.5)
	Type III	7 (8.5)
Symptoms before diagnosis	Asthenia of the four limbs	60 (73.2)
	Decreased muscle tone	53 (64.6)
	Delayed motor function development	58 (70.7)
	Anomalous respiration	12 (14.6)
	Pneumonia or respiratory symptoms	18 (22.0)
	Dysphagia or difficulty in eating	13 (15.9)
	Others	21 (25.6)
Current motor function	Unable to sit unaided	45 (54.9)
	Able to sit unaided but unable to walk independently	9 (11.0)
	Able to walk independently but with varying degrees of asthenia	3 (3.6)
	Able to walk independently	4 (4.9)
	Others	21 (25.6)
Prevalent comorbidities	Respiratory problems	25 (31.6)
	Alimentary problems	38 (48.1)
	Psychological problems	26 (32.9)
	Skeletal problems	63 (79.7)
	Others	11 (13.9)
Medical treatments	Yes	79 (96.3)
	No	3 (3.7)
Rehabilitation treatments	Yes	71 (86.6)
	No	11 (13.4)

### Current medical treatments

#### Treatment patterns and medication utilization

Treatment patterns and medication utilization for the 79 children with SMA are summarized in Table 3. Among the 79 patients receiving medical interventions, disease-modifying therapies predominated: Nusinersen and Risdiplam were each administered to 70.9% (56/79) of the cohort. Combination regimens were common, with Nusinersen together with Risdiplam constituting 86.5% (32/37) of combination therapies. Furthermore, expectorants/mucolytics (66.7%, 16/24) and antibiotics (58.3%, 14/24) were the most common supportive medications administered.

**Table 3:** Basic information on current medical treatments

Characteristics	Categories	Records [*n* (%)]
Overall medication usage (*n* = 79)	Gene therapy drugs targeting SMN1 (EXG001-307, GC101, Zolgensma)	7 (8.7)
	Nusinersen	56 (70.9)
	Risdiplam	56 (70.9)
	Traditional therapy drug	1 (1.3)
Combination drug therapy (*n* = 37)	Nusinersen + Risdiplam	32 (86.5)
	EXG001-307/GC101 + Nusinersen + Risdiplam	2 (5.4)
	Traditional therapy drug + Nusinersen+Risdiplam	1 (2.7)
	EXG001-307 + Nusinersen	1 (2.7)
	GC101 + Risdiplam	1 (2.7)
Supportive pharmacotherapy (*n* = 24)	Nutritional Supplements	12 (50.0)
	Antibiotics	14 (58.3)
	Expectorants and Mucolytics	16 (66.7)
	Others	3 (4.2)

### DMT utilization patterns

#### Accessibility

For Nusinersen, 71.4% (40/56) reported hospital availability, with 91.1% (51/56) accessing treatment through hospital channels versus 8.9% (5/56) via pharmacies. The number of intrathecal injections ranged from 1 to 8, with 26.8% (15/56) receiving 4 doses (Table 4). Risdiplam showed comparable hospital availability (73.2%, 41/56), though 44.6% (25/56) relied on pharmacy procurement. 26.8% of patients (15/56) demonstrated therapeutic response after 1–2 bottles of medication (Table 5).

**Table 4:** The usage of Nusinersen (*n* = 56)

Questions	Answers	Records [*n* (%)]
Medication available at hospital	Yes	40 (71.4)
	No	16 (28.6)
Medication access channels	Hospital	51 (91.1)
	Pharmaceutical company/pharmacy	5 (8.9)
Effective dose	1 dose	4 (7.1)
	2 doses	2 (3.6)
	3 doses	8 (14.3)
	4 doses	15 (26.8)
	5 doses	5 (8.9)
	6 doses	5 (8.9)
	7 doses	3 (5.4)
	8 doses	2 (3.6)
	Others	12 (21.4)
Therapeutic response metrics	Independent grasps	23 (41.1)
	Kicks	22 (39.3)
	Head control	25 (44.6)
	Rolls	21 (37.5)
	Sits	21 (37.5)
	Crawls	12 (21.4)
	Stands	8 (14.3)
	Walks	8 (14.3)
	Others	12 (21.4)
Adverse reactions	Gastrointestinal reactions	1 (1.8)
	Infections	2 (3.6)
	Various types of rashes	1 (1.8)
	Others	4 (7.1)
	No adverse reaction occurred	48 (85.7)
Implementation challenges	Medication is expensive	25 (52.1)
	Hospital treatment is troublesome	22 (45.8)
	Intrathecal injection is challenging	28 (58.3)
	Excessive radiation exposure	6 (12.5)
	Others	3 (6.3)

**Table 5:** The usage of Risdiplam (*n* = 56)

Questions	Answers	Records [*n* (%)]
Medication available at hospital	Yes	41 (73.2)
	No	15 (26.8)
Medication access channels	Hospital	31 (55.4)
	Pharmaceutical company/pharmacy	25 (44.6)
Effective dose	1–2 bottles	15 (26.8)
	3–4 bottles	10 (17.9)
	5–6 bottles	11 (19.6)
	7–8 bottles	5 (8.9)
	Others	15 (26.8)
Therapeutic response metrics	Independent grasps	21 (37.5)
	Kicks	18 (32.1)
	Head control	22 (39.3)
	Rolls	16 (28.6)
	Sits	20 (35.7)
	Crawls	6 (10.7)
	Stands	11 (19.6)
	Walks	7 (12.5)
	Others	18 (32.1)
Adverse reactions	Gastrointestinal reactions	3 (5.4)
	Various types of rashes	5 (8.9)
	Fever	4 (7.1)
	Urinary tract infection	1 (1.8)
	Joint pain	2 (3.6)
	Darkening or yellowing of the skin	9 (16.1)
	Others	2 (3.6)
	No adverse reactions occurred	37 (66.1)
Implementation challenges	Medication is expensive	20 (54.1)
	Preparation is troublesome	11 (29.7)
	Feeding difficulties	2 (5.4)
	Drug storage is challenging	19 (51.4)
	Others	6 (16.2)

#### Patient-reported outcomes and tolerability

Based on caregiver-reported perceptions, no statistically significant differences were observed between Nusinersen and Risdiplam in achieving significant clinical improvement (64.3% vs. 58.9%, *p* = 0.445) (Table 6). Among patients treated with Nusinersen, 44.6% (25/56) achieved head control, 41.1% (23/56) achieved independent grasping ability, and 37.5% (21/56) achieved the ability to sit independently. In the Risdiplam-treated group, 39.3% (22/56) attained head control, 37.5% (21/56) achieved grasping ability, and 35.7% (20/56) achieved sitting ability (Tables 4 and 5).

**Table 6:** Comparison between Nusinersen and Risdiplam (*n* = 56)

Items	Nusinersen (*n* = 56)	Risdiplam (*n* = 56)	*p* values
Medication efficacy, n (%)			
Significant changes	36 (64.3)	33 (58.9)	0.445
No significant changes	8 (14.3)	13 (23.2)	
Poor efficacy	5 (8.9)	2 (3.6)	
Indeterminable	7 (12.5)	8 (14.3)	
Medication costs, n (%)			
High	45 (80.4)	42 (75.0)	0.791
Normal	8 (14.3)	10 (17.9)	
Others	3 (5.4)	4 (7.1)	
Adverse reactions, n (%)			
Yes	8 (14.3)	19 (33.9)	0.015
No	48 (85.7)	37 (66.1)	
Sustained medical costs over the long term, n (%)			
Yes	16 (28.6)	13 (23.2)	0.67
No	15 (26.8)	19 (33.9)	
Indeterminable	25 (44.6)	24 (42.9)	

Caregivers reported a higher incidence of perceived adverse events (AE) with Risdiplam compared with Nusinersen (33.9% vs. 14.3%, *P* = 0.015; Table 6). The Risdiplam-treated group reported skin discoloration (16.1%, 9/56) and rashes (8.9%, 5/56) as the most frequently observed AEs, whereas the Nusinersen-treated group with 85.7% (48/56) of patients experiencing no AEs (Tables 4 and 5).

#### Implementation challenges and long-term sustainability

The most frequently reported challenge for Nusinersen was intrathecal injection logistics (58.3%, 28/56), while for Risdiplam, drug storage difficulties were most common (51.4%, 19/56) (Tables 4 and 5).

Cost constraints were reported by 80.4% (45/56) of Nusinersen and 75.0% (42/56) of Risdiplam, with no significant intergroup differences (*p* = 0.791; Table 6). Sustained medical costs over the long term remained indeterminable for 44.6% (25/56) of Nusinersen and 42.9% (24/56) of Risdiplam (Table 6).

#### Rehabilitative intervention utilization

A total of 71 children with SMA were included in the analysis of rehabilitation management status (Table 7). The most prominent rehabilitation interventions included muscle motor function training, which was utilized in the vast majority of patients (93.0%, 66/71), and the use of a standing frame for stretching (66.2%, 47/71). Medical institutions were the most common rehabilitation setting (42.3%, 30/71), while nearly half of the patients (47.9%, 34/71) required respiratory and sputum management assistance, and 43.7% (31/71) of children received no specific interventions for swallowing or nutrition.

**Table 7:** Rehabilitation Management Status (*n* = 71)

Characteristics	Items	Records [*n* (%)]
Rehabilitation treatment locations	Home	15 (21.1)
	Medical institutions	30 (42.3)
	Rehabilitation centers	26 (36.6)
Exercise training	Anti-gravity/Resistance training	34 (47.9)
	Aquatic exercise training	27 (38.0)
	Assisted walking training	23 (32.4)
	Muscle motor function training	66 (93.0)
	Others	2 (2.8)
Use of assistive devices	Standing frame	47 (66.2)
	Wheelchair	14 (19.7)
	Transfer board	1 (1.4)
	Traction device	4 (5.6)
	Others	9 (12.7)
	Not used	12 (16.9)
Respiratory and sputum management	Cough assist machine, sputum excretion machine, suction machine	34 (47.9)
	Invasive/Non-invasive ventilator	8 (11.3)
	No respiratory issues	29 (40.8)
	Others	10 (14.1)
Swallowing and nutrition management	Swallowing training	27 (38.0)
	Oral sensory stimulation	18 (25.4)
	Assisted methods such as oral/nasal tube feeding or gastrostomy	7 (9.9)
	Nutritional supplements	10 (14.1)
	Others	2 (2.8)
	None	31 (43.7)

#### Nursing management

A total of 82 SMA caregivers were evaluated for nursing management status, revealing notable insights into their training needs and knowledge acquisition (Table 8). The majority of caregivers (98.8%) acknowledged the importance of nursing, yet a smaller fraction (11.0%, 9/82) had received formal nursing training. There was a pronounced demand for home nursing skills (92.7%, 76/82). Social media platforms, particularly TikTok (43.8%, 32/73) and official organizational accounts (41.1%, 30/73), were the primary channels for acquiring nursing knowledge.

**Table 8:** Nursing management status

Characteristics	Items	Records [*n* (%)]
Importance of Nursing (*n* = 82)	Yes	81 (98.8)
	No	1 (1.2)
Nursing Training Received (*n* = 82)	Yes	9 (11.0)
	No	73 (89.0)
Nursing Training Needs (*n* = 82)	Home nursing skills	76 (92.7)
	Family emergency measures	50 (61.0)
	Gastrostomy and nasogastric tube care	6 (7.3)
	Others	5 (6.1)
Forms of Nursing Training (*n* = 9)	Oral education	7 (77.8)
	Expert lectures	3 (33.3)
	Distribute brochures	1 (11.1)
	Rehabilitation training courses	4 (44.4)
	Others	1 (11.1)
Channels for Acquiring Nursing Knowledge (*n* = 73)	TikTok	32 (43.8)
	Rednotes	22 (30.1)
	Weibo	5 (6.9)
	Official account	30 (54.8)
	Hospital	13 (17.8)
	Patient groups	15 (20.5)

#### Clinical trial engagement perceptions

Awareness of ongoing clinical trials for SMA therapeutics was high among caregivers, with 84.1% (69/82) reporting familiarity with such studies, and 74.4% (61/82) viewed these trials as an essential part of treatment. While 58.5% (48/82) expressed willingness for their child to participate, a minimal proportion (8.5%, 7/82) had actual prior participation experience (Table 9).

**Table 9:** Perspectives of respondents on drug clinical trials (*n* = 82)

Questions	Answers	Records [*n* (%)]
Are you aware that there are currently clinical trials for SMA medications being conducted?	Yes	69 (84.1)
	No	13 (15.9)
Are you willing for your child to participate in clinical trials for medications?	Yes	48 (58.5)
	No	34 (41.5)
What is your perspective on the role of clinical trials in SMA treatment?	It is an essential part of SMA treatment	61 (74.4)
	Reserved attitude, need more information	18 (22.0)
	Believe it carries risks	1 (1.2)
	Others	2 (2.4)

#### Medication preferences and economic considerations in SMA caregivers

The vast majority of caregivers prioritized significant efficacy (97.6%, 80/82) and affordable cost (81.7%, 67/82) as the most desired drug attributes. Oral administration was the most accepted route (58.5%, 48/82), and sweet, fruit-flavored medications were strongly preferred (75.6%, 62/82). Furthermore, cost was identified as a significant factor influencing treatment choices by 95.1% (78/82) of respondents (Table 10).

**Table 10:** Medication preferences and economic considerations (*n* = 82)

Questionnaires	Records [*n* (%)]
What route of administration do you think is more acceptable for children?	
Intravenous injection	21 (25.6)
Intrathecal injection	3 (3.7)
Intramuscular injection	6 (7.3)
Oral administration	48 (58.5)
Inhalation administration	3 (3.7)
Others	1 (1.2)
What types of drug formulations do you think are more acceptable for children?	
Tablets/Capsules	8 (9.8)
Solution	25 (30.5)
Drops	26 (31.7)
Injection	9 (11.0)
Suppository	1 (1.2)
Effervescent tablets	4 (4.9)
Chewable tablets	8 (9.8)
Others	1 (1.2)
What flavors of medication do you think are more acceptable for children?	
Sweet tasting	11 (13.4)
Sweet tasting with fruit flavor	62 (75.6)
Tasteless	9 (11.0)
Do you think the price will influence patients’ choice of medication?	
Yes	78 (95.1)
No	4 (4.9)
Do you think the current price of SMA treatment drugs is acceptable?	
Yes	38 (46.3)
No	44 (53.7)
What kind of medication do you wish for your child to use?	
Easy to use	66 (80.5)
Affordable	67 (81.7)
Significant efficacy	80 (97.6)
Short treatment duration	61 (74.4)
Others	5 (6.1)

## Discussion

As an exploratory survey, this caregiver-centered study provides a descriptive, hypothesis-generating overview of disease burden, treatment patterns, and unmet needs among Chinese SMA patients and caregivers. Our findings highlight several critical challenges, including: (1) limited accessibility to prenatal/newborn SMA genetic screening, (2) substantial economic barriers impeding access to DMTs, (3) significant unmet needs in rehabilitation and nursing care, and (4) significant gaps in caregiver training. These observations point to urgent priorities for healthcare policy reform and clinical practice optimization. By addressing the notable gap in real-world, patient- and caregiver-reported data within China, this study lays a foundation for refining diagnostic and therapeutic approaches, informing evidence-based support policies, and guiding future targeted research.

Early diagnosis and therapeutic intervention significantly improve the prognosis of SMA patients [19]. Prenatal diagnosis reduces the birth of affected infants by enabling legal pregnancy termination in cases of severe genetic conditions. Systematic newborn or pediatric screening facilitates early detection, diagnosis, and intervention to attenuate disease severity [22]. Current evidence confirms that prenatal screening substantially decreases SMA birth rates, while newborn screening programs ensure timely diagnosis and access to DMTs [23]. In the United States, screening is available in most states [24, 25]; in Canada, programs are operational in several provinces [26, 27]; and a successful pilot in Southern Belgium led to an official program [28]. Some countries also perform preconception screening [29, 30]. These initiatives provide scalable models. However, China has yet to establish nationwide SMA screening, despite an estimated incidence of approximately 1 in 9,788 newborns. Future efforts should focus on incorporating SMA into the national neonatal screening program, supported by medical insurance coverage, to reduce diagnostic delays [31, 32].

The high utilization rates of DMTs-Nusinersen (70.9%) and Risdiplam (70.9%)-reflect China’s rapid progress in expanding access, marked by their approval and inclusion in the Chinese National Reimbursement Drug List [12]. However, 53.7% of caregivers reported treatment costs as unaffordable, likely due to a complex financial burden encompassing medication, comorbidity management, rehabilitation, and lost income. Our findings are consistent with global surveys indicating substantial financial burdens among SMA families worldwide [26, 33]. These findings echo the global discourse on orphan drug pricing, where high development costs and small patient populations create ethical dilemmas between innovation and equity. Research reveals systemic challenges within the orphan drug market [34]. Several European nations have implemented governmental interventions to contain pricing and promote cost-effective use [34], and Spain has established a patient-centered reimbursement assessment framework [35]. In recent years, China has introduced policies accelerating review and reimbursement for orphan drugs. Building on international practices, China should implement value-based payment models and streamline reimbursement mechanisms to reduce catastrophic health expenditures [12].

Beyond drug cost pressures, significant logistical challenges also exist. Most caregivers report that Nusinersen requires repeated hospitalizations for intrathecal administration via lumbar puncture, posing substantial convenience burdens, with increased technical difficulty in patients with spinal deformities [36]. Risdiplam offers oral convenience but faces practical limitations in prescription renewal and cold-chain storage. These findings align with prior literature on high-cost therapies in resource-limited settings, where logistical complexities hinder access [37]. Addressing these issues may involve exploring the establishment of intrathecal administration centers within the tiered healthcare system or developing a temperature-controlled delivery network for community pharmacies.

There is no definitive evidence of benefit from DMT combination therapy, though its use is growing globally [19, 25]. In our study, the most prevalent combination therapy was concurrent administration of Nusinersen and Risdiplam. Potential drivers include: the clinical imperative in severe SMA, China’s unique reimbursement landscape enabling combination therapy, and evidence-constrained parental preference for rapid response. Establishing a national treatment registry is critical to monitor long-term benefit-risk ratios, particularly for potential synergistic hepatorenal and neurophysiological toxicities, thereby generating practice-changing evidence.

A previous meta-analysis showed Nusinersen and Risdiplam had similar AE rates versus placebo but omitted direct comparison [38]. We observed a higher incidence of caregiver-reported AEs with Risdiplam relative to Nusinersen, including skin discoloration in 9 patients-a reaction not widely reported previously [38, 39]. Given the small sample size of our study, this finding may represent a chance occurrence. Further investigation with larger cohorts is required to determine whether this effect is causally related to the medication. Moreover, these observations are derived solely from caregiver-reported experiences and should therefore be interpreted with caution, as they do not constitute objective clinical evidence of differential drug safety or efficacy.

Our study found that rehabilitation and nursing care represent significant unmet needs. Our study found that rehabilitation and nursing care represent significant unmet needs. Despite high participation rates, critical gaps exist: 40.8% of patients received no respiratory management (increasing pneumonia risk), 43.7% lacked swallowing/nutritional support (potentially leading to growth retardation), and only 32.4% engaged in assisted walking training (limiting functional compensation). These gaps relate directly to SMA risks including respiratory insufficiency, dysphagia, and malnutrition [40–42]. In nursing, while 98.8% of caregivers recognize its importance, 89.0% have never received formal training. Their most urgent needs center on home nursing skills and family emergency measures. In the absence of formal training, caregivers predominantly rely on short-video platforms and social media for health information-a practice raising significant concerns about the reliability and systematic rigor of these sources. While such new media (including social platforms, short videos, and professional health communication channels) play an increasingly vital role in the health literacy of SMA patients and families [43], establishing a standardized, accessible caregiver training system remains essential for improving home-based management quality. To bridge this gap, we propose developing hospital-led digital education platforms, exemplified by mini-program courses and online skills assessments, that leverage digital accessibility while ensuring medical authority.

The present study has several limitations that should be considered. The cross-sectional design precludes causal inference, and the self-reported data are susceptible to recall and social desirability biases. Detailed cost data, standardized quality-of-life metrics, and objective motor function scores were not collected. Type IV (adult-onset) SMA patients were not included, and the lack of systematic age data may have led to an overrepresentation of younger patients. The generalizability of our findings is primarily constrained by the sampling method. Our convenience sample, recruited via online patient communities, likely over represents caregivers who are digitally engaged, actively seeking information, and potentially from higher socioeconomic backgrounds. This introduces a selection bias that intersects with China’s geographic disparities. Consequently, our sample may predominantly reflect the experiences of families in urban and eastern developed regions, while underrepresenting those in rural, central, western, or medically underserved areas. Compared to the broader epidemiological context of SMA in China—where the estimated incidence is approximately 1 in 9,788 newborns—our cohort’s alignment with the expected predominance of severe pediatric-onset types (I-III) is reassuring for internal validity. However, this sampling approach likely leads to an overestimation of real-world DMT utilization and awareness of novel treatments, as it captures a more “activated” segment of the caregiver population. It may also underestimate the true prevalence of systemic access barriers faced by harder-to-reach groups. Furthermore, while the questionnaire demonstrated face and content validity through a multi-stage development process involving stakeholder feedback, its psychometric properties (e.g., internal consistency) were not formally assessed.

## Conclusions

Findings from this exploratory caregiver survey underscore several persistent challenges in managing SMA in China, including prohibitive treatment costs, logistical barriers to drug administration, gaps in multidisciplinary supportive care, and a widespread lack of formal caregiver training. These results point to urgent systemic priorities: policy reforms to ensure sustainable drug access, the implementation of standardized care protocols, and the establishment of structured family education programs. To advance the field, future research should focus on: (1) longitudinal assessments of the long-term real-world outcomes of disease-modifying therapies; (2) the development of scalable, integrated care and training models for underserved regions; and (3) health economic analyses to optimize China’s multi-tiered medical security framework. Addressing these areas will be crucial for improving health outcomes and quality of life for all individuals with SMA in China.

## Supplementary Information

Supplementary Material 1.

Supplementary Material 2.

## Data Availability

The original contributions presented in the study are included in the article, further inquiries can be directed to the corresponding authors.
