# Supplementary figures and images for: Real-world treatment patterns and unmet needs in spinal muscular atrophy: a caregiver-centric survey study from China
Source: BMC Neurol. 2026 Feb 27;26:219. doi: 10.1186/s12883-026-04774-z (PMC13049836; doi:10.1186/s12883-026-04774-z)

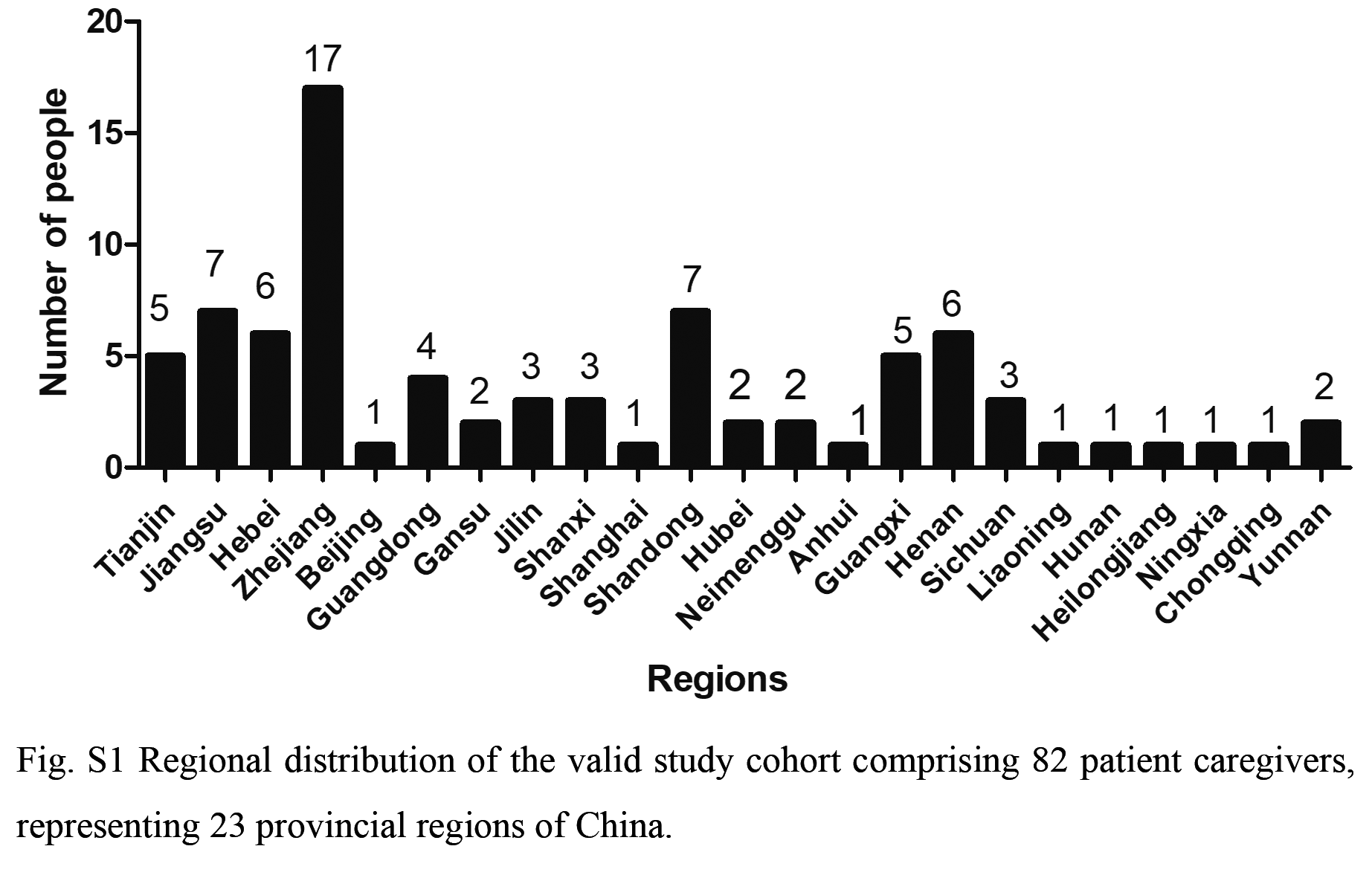

Supplement: Supplementary file 1 — Supplementary Material 1. [file 12883_2026_4774_MOESM1_ESM.tif]
