# Supplementary material for: Real-world treatment patterns and unmet needs in spinal muscular atrophy: a caregiver-centric survey study from China
Source: BMC Neurol. 2026 Feb 27;26:219. doi: 10.1186/s12883-026-04774-z (PMC13049836; doi:10.1186/s12883-026-04774-z)
Supplement: Supplementary file 2 — Supplementary Material 2. [file 12883_2026_4774_MOESM2_ESM.docx]

**Dear Sir/Madam,**

**Greetings,**

We are the Office of the Clinical Trial Institute at the Children's Hospital, Zhejiang University School of Medicine, and the National Clinical Research Center for Child Health. We sincerely invite you to participate in a survey on medication use and treatment needs among patients with Spinal Muscular Atrophy (SMA), which has been approved by our institution. This survey aims to understand the real-world experiences of SMA patients and their guardians regarding daily medication, rehabilitation, and care processes. Centered on SMA patients and their guardians, the questionnaire is designed to gather and analyze information across three dimensions: demographic characteristics, medication usage, and needs assessment. The goal is to understand the actual medication practices of SMA patients and their needs and expectations during treatment. This research will help us gain a more detailed understanding of this rare disease community, better listen to the voices of patients and families, contribute to advancing research in the SMA field, truly achieve patient-centered care, and provide new insights and methods for future drug development and clinical diagnosis and treatment.

Completing this questionnaire will take approximately 8-10 minutes. The process is anonymous from distribution to completion. All collected data will be used solely for academic research purposes. Your information will not be disclosed to any organization or third party and will absolutely not be used for any commercial purposes. Your personal information will be kept strictly confidential. Please feel assured in providing your responses. There are no right or wrong answers to the questions; please answer as truthfully and accurately as possible based on your actual situation. Furthermore, participation in this study is entirely voluntary. You may refuse to participate without giving a reason, and this decision will not affect your participation in any other research.

**Participant Consent Statement:**

I have read the above information regarding this study and fully understand the potential risks and benefits associated with participation. I voluntarily agree to participate in the study described herein.

□ Agree □ Disagree

**1. Demographic Information**

1. Which city do you reside in? (Province + City)

Free text entry

1. What is your child's sex?

A Male

B Female

1. What is your relationship to the child?

A Father

B Mother

C Other guardian

1. What was your child's age (in months or years) at the time of SMA diagnosis?

Free text entry

1. What is your child's SMA type?

A Type I SMA

B Type II SMA

C Type III SMA

D Type IV SMA

1. Was your child's SMA type confirmed by genetic testing?

A Yes

B No

1. Were you aware that SMA genetic screening can be performed during pregnancy? (Selecting 'Yes' proceeds to Q9; Selecting 'No' proceeds to Q8)

A Yes

B No

1. Do you believe awareness regarding prenatal SMA genetic screening should be strengthened? (After selection, proceed to 'Medication Usage' section)

A Yes

B No

1. Did the child's biological mother undergo SMA genetic screening during pregnancy? (Selecting 'Yes' proceeds to Q10; Selecting 'No' proceeds to Q11)

A Yes

B No

1. Why was prenatal SMA genetic screening chosen? (After selection, proceed to 'Medication Usage' section)

A Doctor's recommendation

B Recommendation from other pregnant women/friends

C Personal knowledge/initiative

D Others(please specify)

1. Why was prenatal SMA genetic screening not chosen? (After selection, proceed to 'Medication Usage' section)

A Perceived as unnecessary

B Out-of-pocket cost, not reimbursable by insurance

C Others(please specify)

**2. Medication Usage**

1. What symptoms did your child present with prior to diagnosis? (Multiple choice)

A Asthenia of the four limbs

B Decreased muscle tone

C Delayed motor function development

D Anomalous respiration

E Pneumonia or respiratory symptoms

F Dysphagia or difficulty in eating

G Others(please specify)

1. What challenges do you perceive exist in the treatment of SMA? (Multiple choice)

A Difficulty in diagnosis

B Limited available medications

C High medical costs

D Challenges in rehabilitation therapy

E Others(please specify)

1. Is your child currently receiving pharmacological treatment for SMA? (Selecting 'Yes' proceeds to Q5; Selecting 'No' proceeds to Q2 in 'Needs - Medication' section)

A Yes

B No

1. Which medication(s) is your child currently using for treatment? (Multiple choice)

A Gene therapy drugs targeting SMN1(EXG001-307, GC101, Zolgensma)

B Nusinersen

C Risdiplam

D Drugs aiming to increase full-length SMN protein levels (e.g., Valproic acid, Celecoxib, etc.)

E Neuroprotective drugs, muscle activators, and others (e.g., Olesoxime, Albuterol, etc.)

F Others(please specify)

1. How would you assess the efficacy of your child's current medication(s)?

A Significant improvement

B No significant change

C Poor efficacy

D Uncertain

1. Besides motor abilities, which of the following areas pose challenges in your child's daily life? (Multiple choice)

A Respiratory problems

B Alimentary problem

C Psychological problem

D Skeletal problems (e.g., scoliosis, contractures)

E Others(please specify)

1. Besides SMA-specific medications, is your child using other drugs for managing complications (e.g., pneumonia, scoliosis, etc.)? (Selecting 'Yes' proceeds to Q9; Selecting 'No' proceeds to 'Nusinersen' section Q1)

A Yes

B No

1. Which medications has your child used for managing disease complications?

A Nutritional supplements

B Antibiotics

C Expectorants and Mucolytics

D Analgesics / Pain medication

E Psychotropic drugs / Medication for psychological care

F Others(please specify)

**Nusinersen**

1. Has your child ever used Nusinersen? (Selecting 'No' proceeds to 'Risdiplam' section)

A Yes

B No

1. Is Nusinersen available at your local hospital?

A Yes

B No

1. How did you access Nusinersen for your child?

A Purchased through the hospital

B Purchased from pharmaceutical company/pharmacy

C Participation in a clinical trial

D Others(please specify)

1. Date of your child's first intrathecal injection of Nusinersen?

Free text entry (likely date)

1. How many intrathecal injections of Nusinersen has your child received to date?

A 1 injection

B 2 injections

C 3 injections

D 4 injections

E Others (please specify)

1. After which injection did your child show significant functional improvement (e.g., ability to roll over, sit independently, etc.)?

A 1st injection

B 2nd injection

C 3rd injection

D 4th injection

E Others (e.g., later injection, not yet)

1. How would you assess the efficacy of Nusinersen for your child?

A Significant changes

B No significant changes

C Poor efficacy

D Indeterminable

1. What improvements has your child achieved since using Nusinersen? (Multiple choice)

A Independent grasps

B Kicks

C Head control

D Rolls

E Sits

F Crawls

G Stands

H Walks

I Others (please specify)

1. Did your child experience any adverse reactions after using Nusinersen? (Selecting 'Yes' proceeds to Q10; Selecting 'No' proceeds to Q11)

A Yes

B No

1. Which adverse reactions did your child experience after using Nusinersen? (Multiple choice)

A Dizziness / Headache

B Gastrointestinal reactions

C Bleeding/leakage at injection site

D Infections

E Various types of rashes

F Others (please specify)

1. What challenges have you encountered regarding the use of Nusinersen? (Multiple choice)

A Medication is expensive

B Hospital treatment is troublesome

C Intrathecal injection is challenging

D Excessive radiation exposure

E Others (please specify)

1. What is your perception of the price of Nusinersen?

A Expensive

B Normal

C Inexpensive

D Others (please specify)

1. Is your family able to bear the long-term treatment costs associated with Nusinersen?

A Yes

B No

C Uncertain

1. What do you perceive as the advantages and disadvantages of Nusinersen? (Open-ended)

Advantages:___________________________________________________________ Disadvantages:________________________________________________________

**Risdiplam**

1. Has your child ever used Risdiplam? (Selecting 'No' proceeds to 'Needs Assessment' section)

A Yes

B No

1. Is Risdiplam available at your local hospital?

A Yes

B No

1. How did you access Risdiplam for your child?

A Purchased through the hospital

B Purchased from pharmaceutical company/pharmacy

C Participation in a clinical trial

D Others(please specify)

1. Date of your child's first dose of Risdiplam?

Free text entry (likely date)

1. After how many bottles of Risdiplam did your child show significant functional improvement (e.g., ability to roll over, sit independently, etc.)?

A 1-2 bottles

B 3-4 bottles

C 5-6 bottles

D 7-8 bottles

E Others (please specify)

1. How would you assess the efficacy of Risdiplam for your child?

A Significant changes

B No significant changes

C Poor efficacy

D Indeterminable

1. What improvements has your child achieved since using Risdiplam? (Multiple choice)

A Independent grasps

B Kicks

C Head control

D Rolls

E Sits

F Crawls

G Stands

H Walks

I Others(please specify)

1. Did your child experience any adverse reactions after using Risdiplam? (Selecting 'NO' proceeds to Q10)

A Yes

B No

1. Which adverse reactions did your child experience after using Risdiplam? (Multiple choice)

A Gastrointestinal reactions (diarrhea, nausea, etc.)

B Various types of rashes

C Headache

D Fever

E Urinary tract infection

F Joint pain

G Others (please specify)

1. What challenges have you encountered regarding the use of Risdiplam? (Multiple choice)

A Preparation is troublesome

B Feeding difficulties

C Drug storage is challenging

D Medication is expensive

E Others (please specify)

1. What is your perception of the price of Risdiplam?

A Expensive

B Normal

C Inexpensive

D Others (please specify)

1. Is your family able to bear the long-term treatment costs associated with Risdiplam?

A Yes

B No

C Uncertain

1. What do you perceive as the advantages and disadvantages of Risdiplam? (Open-ended)

Advantages:___________________________________________________________ Disadvantages:________________________________________________________

**3. Needs Assessment**

**(1) Medication**

1. What is/are the route(s) of administration for your child's current medication(s)? (Multiple choice)

A Intravenous injection

B Oral administration

C Intrathecal injection

D Other (please specify)

1. What route of administration do you think is more acceptable for children?

A Intravenous injection

B Intrathecal injection

C Intramuscular injection

D Oral administration

E Inhalation administration

F Transdermal administration

G Others(please specify)

1. What types of drug formulations do you think are more acceptable for children?

A Tablets/Capsules

B Solution

C Drops

D Injection

E Suppository

F Effervescent tablets

G Chewable tablets

H Others(please specify)

1. What flavors medication do you think are more acceptable for children?

A Sweet tasting

B Sweet tasting with fruit flavor

C Sour tasting

D Tasteles

E Others(please specify)

1. Do you think the price will influence patients' choice of medication?

A Yes

B No

1. Do you think the current price of SMA treatment drugs is acceptable? (Selecting 'Yes' proceeds to Q8; Selecting 'No' proceeds to Q7)

A Yes

B No

1. What measures do you think should be taken by the government/society to reduce SMA drug prices? (Multiple choice)

A Increase funding for drug research and development, encourage corporate R&D

B Establish and improve medical security systems, expand reimbursement rates

C Establish special assistance funds to help patients from financially disadvantaged families

D Others (please specify)

1. What kind of medication do you wish for your child to use? (Multiple choice)

A Easy to use

B Affordable

C Significant efficacy

D Short treatment duration

E Others (please specify)

1. Are you aware that there are currently clinical trials for SMA medications being conducted?

A Yes

B No

1. What is your perspective on the role of clinical trials in SMA treatment?

A It is an essential part of SMA treatment

B Reserved attitude, need more information

C Believe it carries risks

D Others (please specify)

1. Are you willing for your child to participate in clinical trials for medications? (Selecting 'Yes' proceeds to Q12; Selecting 'No' proceeds to Q13)

A Yes

B No

1. What are your reasons for being willing to let your child participate?

Free text entry

1. What are your reasons for not being willing to let your child participate?

Free text entry

**(2) Rehabilitation**

1. What is your child's current functional status?

A Unable to sit unaided

B Able to sit unaided but unable to walk independently

C Able to walk independently but with varying degrees of asthenia

D Able to walk independently

E Other (please specify)

1. Has your child received rehabilitation therapy? (Selecting 'Yes' proceeds to Q3; Selecting 'No' proceeds to 'Care' section)

A Yes

B No

1. Where does your child receive rehabilitation therapy?

A Home

B Medical institutions (e.g., hospital)

C Rehabilitation centers

D Others (please specify)

1. What does your child's rehabilitation therapy consist of? (Multiple choice)

A Respiratory and sputum management

B Stretching exercises

C Motor training / Physiotherapy
D Swallowing and nutrition management

E Use of assistive devices / Orthotics

F Others (please specify)

1. Which methods have you used to help manage your child's respiratory issues? (Multiple choice)

A Mechanical insufflation-exsufflation (Cough assist), Chest physiotherapy devices, Suction machines

B Invasive/Non-invasive ventilation

C Tracheostomy

D Others (please specify)

1. Which assistive devices has your child used to aid mobility training/improvement? (Multiple choice)

A Standing frame

B Wheelchair

C Transfer board

D Traction device

E Others (please specify)

1. Which methods has your child used for swallowing and nutritional management? (Multiple choice)

A Swallowing training

B Oral sensory stimulation

C Assisted methods such as oral/nasal tube feeding or gastrostomy

D Nutritional supplements

E Others (please specify)

1. What types of motor training (Exercise training) has your child undergone?

A Anti-gravity/Resistance training

B Aquatic exercise training

C Assisted walking training

D Muscle motor function training

E Others (please specify)

**(3) Care / Nursing Management**

1. Do you believe nursing/care is important in SMA management?

A Yes

B No

1. Have you received professional care training? (Selecting 'Yes' proceeds to Q3; Selecting 'No' proceeds to Q4)

A Yes

B No

1. What type of care training have you received?

A Oral education

B Expert lectures

C Distribute brochures

D Rehabilitation training courses

E Others (please specify)

1. Through which channels do you acquire knowledge about SMA care? (Multiple choice)

A Douyin (TikTok)

B Xiaohongshu (Rednotes)

C Official account

D Newspapers

E Weibo

F Others (please specify)

1. What are your needs regarding care for this disease? (Multiple choice)

A Home nursing skills training

B Family emergency measures

C Gastrostomy and nasogastric tube care

D Others (please specify)

1. What challenges or problems do you perceive exist in the care for this disease?

Free text entry
